# Disablement Benefit—The Last of the Benefits to Come into Operation

**Published:** 1914-07-25

**Authors:** 


### NATIONAL INSURANCE ACT DEVELOPMENTS.

### Disablement Benefit—the Last of the Benefits to Come into Operation.

The National Insurance Act of 1911 has now been in operation for two years. The fact is important not merely as marking the second anniversary of a great and far-reaching measure of social legislation, but also because another stage has been reached in the operation of the Act. It is now possible for those who are entitled, by the fact that they have been in insurance for 104 weeks and have paid 104 weekly contributions, to receive disablement benefit. Disablement benefit is the last of the benefits promised by the Act to come into operation, and the commencement of that benefit is a definite stage in the development of the insurance scheme. In order that an insured person may be entitled to claim disablement benefit he must not only have been in insurance and have actually paid the contributions for the requisite number of 104 weeks, but he must also have exhausted his right to sickness benefit, and remain incapable of work by reason of some specific disease or by bodily or mental disablement.

Disablement benefit is, in fact, in the nature of reduced sickness benefit, and becomes payable when an illness or a series of linked-up illnesses has resulted in the payment of the maximum number of twenty-six weeks' sickness benefit.

Sickness benefit is normally in the case of men 10s. a week, and 7s. 6d. in the case of women; but these rates are subject to reduction in certain circumstances depending upon the age of the insured person, his normal rate of remuneration, and if his contributions are in arrear beyond prescribed limits. Disablement benefit, however, is not subject to these variations, but is at the level rate of 5s. a week both for men and women, the only exception being in the case of aliens. The right to disablement benefit ceases at the age of seventy, when its place will be taken by the national old-age pension, provided the insured person is qualified to receive it.

### Qualifications for the Benefit.

Although disablement benefit must now be considered as in operation its full effect is not likely to be felt for some time. Protracted illnesses of more than twenty-six weeks' duration are very much less common than illnesses of comparatively short duration, and from this fact alone the advent of the new benefit will have little immediate effect on the total sums that are being paid from week to week to insured persons who are incapable of work. Moreover, the benefit can only become payable at once to those who entered insurance at the very commencement of the operation of the Act, and who have paid a contribution for every week since entry. The latter condition implies that contributions must have been paid with the sole object of qualifying for the benefit. No contribution is payable either by the insured person or his employer while sickness benefit is being paid, and in consequence, as the right to sickness benefit must have been exhausted before disablement benefit can be claimed, the insured person must have paid at least twenty-six full contributions of 7d. (6d. in the case of women) from his own resources which need not have been paid, except with the object of qualifying for the disablement benefit. In many cases, where there have been good grounds for supposing that the illness would extend beyond twenty-six weeks, insured persons have doubtless already made provision for the necessary qualification.

But in other cases, where the expectation of a protracted illness has not been so apparent, the necessity for paying the contributions from week to week may have been overlooked. In such event it is possible for the insured person to pay the back contributions in a lump sum by way of arrears—though the stamps must not be affixed to a special arrears card, but to an ordinary contribution card. It will, of course, be a matter for each individual to decide whether it is to his advantage to pay the qualifying contributions or not. If the illness is likely to continue for at least another three weeks, it would in general be to the insured person's advantage to pay the contributions to complete the qualification.

Besides the contributions that are unpaid owing to the receipt of sickness benefit it may happen that there are arrears of contribution due to periods of unemployment. If these arrears are due for any weeks within the first year after the Act came into operation, they will have to be paid for the purpose of qualification at the full rate, because unpaid contributions within the first year were specifically excluded by the Act from counting as arrears. If, however, the unemployment has occurred during the past year the arrears can be made good by paying up the portion applicable to the insured person only, as the payment of the employer's share is now excused in such cases.

### The Heavy Liability on Approved Societies.

In course of time, as more and more insured persons become entitled by the duration of their insurance and the number of contributions paid to the right to disablement benefit, and likewise as their right to sickness benefit becomes exhausted, there will be an ever-increasing number of persons in receipt of the disablement allowance. With the increase in the number of persons in receipt of the allowance the charge on the approved societies will increase, and extreme vigilance will have to be maintained to ensure the financial ability of the societies to meet this charge on their funds. Due allowance has, of course, been made in the contributions for the liabilities anticipated under this benefit, but it is known that the actuarial tables upon which the estimates were based were less reliable as an index of protracted sickness than for illnesses of short duration. It may well happen that in total the actuarial estimates on account of this benefit will be justified, at any rate as regards male insured persons, even if they do not actually provide a margin over and above the gross liabilities as they accrue. But owing to the method adopted of administering the Act through separate and independent societies it is apparent that many societies will find it difficult with their present resources to meet the liability that will be imposed upon them by the payment of disablement benefit. In many cases, and particularly in the heavy occupations, it will almost certainly be found that a large proportion of those who become entitled to the benefit will remain permanently incapacitated for work, and the allowance will therefore be in the nature of an annuity terminating on the attainment of the age of seventy or on earlier death.

### Societies in the Heavy Trades.

An actuarial training is not required to perceive that a liability of this description is of an onerous character, and that it is greater than the liability, when age and other conditions are taken into account, for a sickness benefit of double the amount weekly which can only be claimed by a person, now in normally good health, when he is rendered incapable of work by sickness. Societies, therefore, which are mainly or exclusively confined to workers in the heavy trades will most probably experience an undue share of the protracted illness which in total, over all the societies, has no doubt been correctly estimated. It is these very societies which are at the present time experiencing high sickness rates, and which also record the highest claims for maternity benefit. The only relief which such societies can at present contemplate is the release from liability for the payment of the benefit, or a portion thereof, when the incapacity is caused by an injury for which the insured member is entitled to claim compensation. Thus it is clear that some modification of the finance of the Act will sooner or later have to be taken in hand.

### A Suggested Equalisation Fund.

This modification is already indicated in the second annual report on National Health Insurance which has just been published, in which the suggestion is made for the institution of an equalisation fund. Space will not allow an exhaustive discussion of this topic here, but it may be pointed out that such a fund would necessitate legislative sanction, and it may be that proposals dealing with the matter will appear in the long-delayed Amendment Bill, which the Chancellor of the Exchequer promised would be introduced as soon as possible after his Budget statement.

From the point of view of the insured person who has unfortunately become permanently incapacitated, it is absolutely essential that there shall be no doubt as to the ability of his society to pay him the benefit in full that is his due. It must not be left to chance that his society may have a deficit at a valuation, and be obliged to reduce the rates of benefit, although that deficit may in part be due to his claims upon the funds. This is a matter that must receive careful attention, for it was one of the objects of the National Insurance scheme to provide a means whereby the insured should be able to draw the benefits to which they had themselves contributed and thus be relieved of the necessity for seeking assistance under the Poor Law. The new benefit is not one that is likely to be attractive to the malingerer, and it is, therefore, all the more necessary that the promised allowance shall be made absolutely secure.

